# TCN-QV: an attention-based deep learning method for long sequence time-series forecasting of gold prices

**DOI:** 10.1371/journal.pone.0319776

**Published:** 2025-05-05

**Authors:** Yishuai Yang

**Affiliations:** 1 College of Management Science, Chengdu University of Technology, Erxianqiao, Chengdu 610059, Sichuan, P.R.China; Air Force Engineering University, CHINA

## Abstract

Accurate prediction of gold prices is crucial for investment decision-making and national risk management. The time series data of gold prices exhibits random fluctuations, non-linear characteristics, and high volatility, making prediction extremely challenging. Various methods, from classical statistics to machine learning techniques like Random Forests, Convolutional Neural Networks (CNN), and Recurrent Neural Networks (RNN), have achieved high accuracy, but they also have inherent limitations. To address these issues, a model that combines Temporal Convolutional Networks (TCN) with Query (Q) and Keys (K) attention mechanisms (TCN-QV) is proposed to enhance the accuracy of gold price predictions. The model begins by employing stacked dilated causal convolution layers within the TCN framework to effectively extract temporal features from the sequence data. Subsequently, an attention mechanism is introduced to enable adaptive weight distribution according to the information features. Finally, the predicted results are generated through a dense layer. This method is used to predict the time series data of gold prices in Shanghai. The optimized model demonstrates a substantial improvement in Mean Absolute Error (MAE) compared to the baseline model, achieving reductions of approximately 5.47% in the least favorable case and up to 33.69% in the most favorable scenario across four experimental datasets. Additionally, the model is tested across different time steps and shows satisfactory performance in long sequence predictions. To validate the necessity of the model components, this paper conducts ablation experiments to confirm the significance of each segment.

## 1 Introduction

As a traditional safe-haven asset, gold price fluctuations directly affect investors’ confidence and market stability. In an uncertain economic environment, gold is widely employed as a hedging tool [1]. Demand for gold often increases during periods of economic turmoil. For example, following the 2016 Brexit referendum, gold prices soared, reflecting market concerns about economic uncertainty and prompting many investors to turn to gold to mitigate risks. Similarly, when the global economy experiences recession or inflationary pressures, the demand for gold tends to increase, consequently driving up its prices. Additionally, gold is often employed as part of a country’s currency and foreign exchange reserves in international trade, playing a crucial role in the financial stability and creditworthiness of various nations. Fluctuations in gold prices directly impact the value of a nation’s foreign exchange reserves, which in turn influences the formulation of monetary policy. Therefore, closely monitoring changes in gold prices enables China to better understand international market dynamics, adjust its economic strategies promptly, maintain financial security, and ensure stable economic development [2].

After analyzing the fluctuations in gold prices and their impacts on the market and economy, researchers have recognized that accurately predicting changes in gold prices is crucial for both investors and policymakers. The price time series data of gold can essentially be considered a specific type of time series data. Time series analysis in the context of pricing can reveal historical trends in price changes and provide insights into potential factors influencing these fluctuations. Moreover, time series models are particularly adept at handling the dynamic characteristics of economic data, making them indispensable for predicting gold prices.

In recent years, scholars have developed various models for predicting metal prices. Traditional time series models, such as Autoregressive Integrated Moving Average (ARIMA), Seasonal Autoregressive Integrated Moving Average (SARIMA), exponential smoothing, vector autoregression (VAR), and state space models, effectively capture basic trends and seasonal fluctuations.

However, these linear models often struggle to explain the complex nonlinear factors that influence metal prices. Consequently, many researchers have shifted their focus to deep learning methods predominantly based on neural networks. For instance, long short-term memory networks (LSTM) [3], gated recurrent units (GRU) [4], and Temporal Convolutional Network (TCN) have proven effective in capturing dependencies and volatility patterns in price fluctuations. In particular, TCNs enhance information flow through dilated causal convolutions, offering significant advantages in training speed and stability [5]. Despite these advancements, recurrent neural networks (RNNs) and their variants face challenges such as gradient vanishing and gradient explosion, which limit their ability to learn long-term dependencies. Similarly, TCNs may still encounter difficulties in managing long-range dependencies, especially in the context of cyclic information transmission.

In addition to traditional neural network models, the application of Transformer architecture in price prediction has attracted increasing attention. Compared with recurrent neural networks, Transformers are more efficient and accurate in processing large-scale datasets [6]. However, their computational complexity is relatively high. Particularly when handling long sequences, it leads to rapidly increasing computational costs and memory requirements, which limits their applicability in resource-constrained environments [7].

Consequently, many researchers have optimized the traditional self-attention mechanism in Transformers and integrated it into recurrent neural network models to enhance predictive performance. However, these optimized models still face the challenge of exponentially increasing computational costs. It is particularly important to develop a model that can effectively extract contextual relationships while ensuring efficient and precise predictions.

To address the challenges of time series prediction, a novel model is introduced that integrates TCNs, Multi-Layer Perceptrons (MLPs), and optimized global attention mechanisms to enhance predictive performance. This model employs dilated causal convolutions stacked in multiple layers, enabling efficient parallel processing of long sequence data.To overcome the limitations of TCNs in capturing long-range dependencies, the output data from the TCN is transformed into a query matrix (Q) and a value matrix (V) through dimensional adjustments, thereby enhancing the model’s ability to interpret long sequences. By utilizing cross-multiplication, a three-dimensional tensor encapsulating contextual relationships is generated. Residual connections link the output and input, forming a complete attention mechanism for the model. This approach not only improves the performance of TCNs in long sequence prediction but also maximizes operational efficiency compared to traditional self-attention mechanisms.

The main contributions of this paper are as follows:

- To enhance the learning effectiveness of metal price time series data, an optimized configuration named TCN-QV is proposed, which combines the TCN with a simplified Transformer structure. This model captures long-term dependencies while maintaining computational efficiency, effectively improving the accuracy of metal price predictions.
- In response to the high computational complexity and insufficient ability of traditional models to extract contextual relationships, the TCN module connected via residuals has been selected. This module employs dilated causal convolution to enhance the network’s capacity to learn spatiotemporal representations.
- To address the exponential increase in computational costs associated with traditional self-attention mechanisms, the QV Attention module has been introduced. This module utilizes a self-attention mechanism to improve the model’s ability to focus on different features while simultaneously reducing time costs.
- Overall, this design-combining TCN and QV Attention-enhances the model’s capacity to capture contextual relationships through the integration of TCN. It also ensures improved feature recognition while maintaining high computational efficiency, enabling more accurate temporal predictions.

The organizational structure of this study is as follows: The second section reviews current research related to time series prediction. The third section details our TCN-QV method. The fourth section introduces the experimental environment and dataset, while the fifth section presents the experimental results and analyses. Finally, the sixth section summarizes the study and includes conclusions and discussions.

## 2 Related work

### 2.1 Time series forecasting

Time series forecasting is a statistical analysis approach that utilizes historical data to predict future events or trends. Time series data consists of observation values arranged in chronological order and typically shows certain patterns of regularity, trends, and seasonality. Throughout history, researchers have proposed and employed various statistical and machine learning methods to improve the predictive performance of models applied to time series data [8]. Scholars have conducted in-depth research based on initial models such as the ARIMA and the SARIMA [9]. For instance, Zhidan Luo et al. enhanced the ARIMA model by incorporating Taylor expansion to capture relevant information [10]. Jinhai Yao et al. developed a combined prediction model integrating ARIMA with information granulation Support Vector Regression (SVR) to forecast stock market index prices and returns [11]. Zhichao He et al. decomposed the original price sequence into Intrinsic Mode Functions (IMF) using Variational Mode Decomposition (VMD), optimized the parameters of VMD using Improved Firefly Algorithm-based Support Vector Regression (IFASSA), and classified the components of VMD and Wavelet Packet Decomposition (WPD) based on the zero-crossing rate. Eventually, ARIMA was utilized for the final prediction [12].

Meanwhile, numerous scholars have shown improved prediction accuracy by combining different types of models with neural networks or integrating various neural network architectures. For example, Rahim Barzegar et al. used boundary correction (BC) Maximum Overlap Discrete Wavelet Transform (MODWT) in combination with a hybrid CNN-LSTM model for water level prediction [13]. Naman Bhoj et al. highlighted the advantages of combination models in prediction accuracy by comparing traditional neural networks with CNN-GRU models [14]. Meanwhile, Xiao Chen et al. employed the LASSO algorithm to obtain optimal feature combinations. Subsequently, they enhanced the information extracted from individual features by integrating it with Cascaded Long Short-Term Memory (Ca-LSTM) technology [15]. These models can generate more accurate future price forecasts by learning patterns from historical data. Consequently, an increasing number of financial institutions and traders are adopting neural network-based prediction models.

Despite significant progress made by researchers in optimizing statistical and machine learning models, traditional deep learning continues to encounter bottlenecks in capturing contextual relationships. While scholars commonly enhance the data capture capabilities of neural network models through the integration of optimization algorithms, there is a noticeable paucity of research aimed at improving predictive performance by focusing on enhancements within the neural network models themselves. Addressing these challenges requires specific adjustments and optimizations to the neural network architectures when confronted with limitations in prediction performance.

### 2.2 Transformer and attention mechanism

With the emergence of the Transformer architecture, its unique self-attention mechanism has demonstrated strong performance and flexibility in image recognition and temporal prediction tasks [16]. Through its self-attention mechanism and parallel processing capabilities, the Transformer can better capture complex patterns in temporal data. As research in this area intensifies, Transformers and their variants are increasingly showing significant application value in time series prediction in various industries and fields [17]. For example, Mehme Burukanli et al. obtained promising results in predicting COVID-19 mutations by using Transformer models combined with Adam optimization algorithms [18]. Among the numerous models developed, many scholars tend to combine Transformers with RNN architectures. For instance, Chengyu Li and Guoqi Qian’s hybrid neural network, the FDG Transformer, integrates GRU, LSTM, and Multi-Head Attention (MHA) components [19]. This model effectively exploits the advantages of both RNNs and Transformers, achieving commendable results in stock prediction. By employing a selective sensing mechanism during context extraction, the model can quickly focus attention on important areas within the data, significantly increasing its sensitivity to relevant information during predictions. Wenjie Lu and Jiazheng Li constructed a CNN for data collection and employed an attention mechanism to assess the impact of stock data at different times on stock prices. The GRU model is also utilized for stock price predictions and proves to be more suitable than traditional neural networks [20]. Jindian Liu, Bo Zhang, and colleagues processed soybean futures price data into multiple IMFs and residual sequences using Ensemble Empirical Mode Decomposition (EEMD) [21]. They developed the NAGU model, which embeds an attention mechanism within the GRU structure and achieves strong results in predicting soybean futures prices.

Compared to traditional machine learning algorithms, the combination of self-attention mechanisms with neural networks offers significant advantages. Despite the variety of models that combine neural networks with attention mechanisms, scholars often focus more on integrating them with GRU or LSTM architectures. Hongfeng Xu, Lei Chai, and others developed a novel stock price trend prediction network based on a reinforcement learning (RL) bidirectional GRU architecture and incorporated an attention mechanism to enhance the model’s prediction accuracy [22]. The introduction of Transformers and self-attention mechanisms has fundamentally transformed the field of deep learning. Their efficiency, flexibility, and scalability serve as the foundation for many cutting-edge research initiatives and applications. However, while scholars frequently enhance model predictive capabilities by using neural network architectures-particularly RNNs-in combination with Transformers to improve sensitivity to time series data, there is relatively little exploration of flexibly combining the self-attention mechanism of Transformers with convolutional neural networks rather than simply parallelizing the entire Transformer model [23]. This gap prompts us to investigate the potential of combining TCNs with attention mechanisms [24].

In summary, individual neural networks still have limitations and struggle to efficiently perform temporal forecasting tasks [25]. Although the parallel processing capabilities of Transformers can be enhanced, redundant calculations continue to impede their efficiency in information extraction [26]. In contrast, modifying neural networks and integrating them with attention mechanisms for effective parallelism appears to be a more promising solution [27]. While researchers frequently employ RNN architectures for prediction tasks, they may overlook the advantages of TCNs in time series forecasting [28]. Therefore, this article proposes the TCN-QV module, which aims to extract contextual information more efficiently, make accurate predictions, and be easy to train.

This study proposes an innovative model that combines a MLP, a TCN, and an attention mechanism to enhance the processing capabilities for time-series data and multidimensional feature tasks. The model initially introduces the DC-Convolution layer, enabling it to effectively capture complex internal dependencies. Subsequently, a global attention mechanism is employed to enable the model to fully utilize information from the entire sequence during decision-making by calculating the query (Q) and value (V), thereby incorporating richer contextual information. To address the computational and memory overhead associated with long time series, a local sliding window method is adopted. This method extracts local features through dilated convolution, reducing computational complexity while preserving the flow of global information.

**Fig 1:**
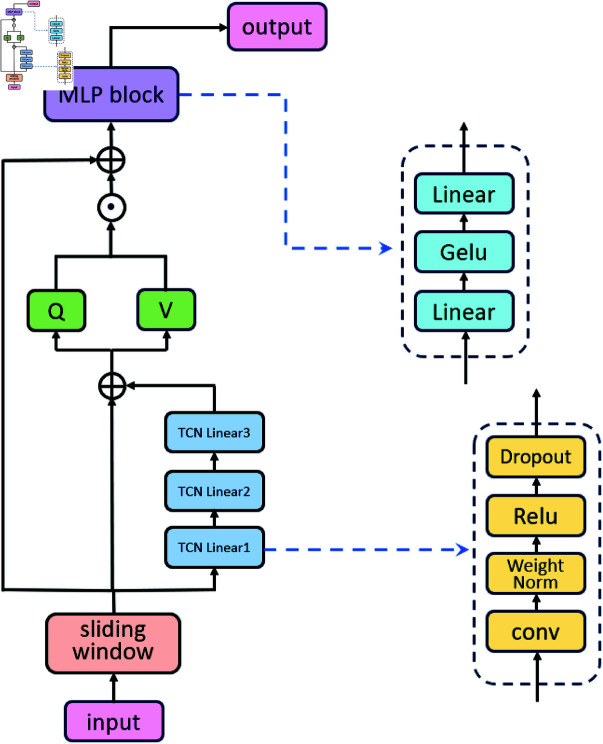
TCN-QV time series prediction model.

Overall, this model increases its adaptability and representational capacity for temporal data through a flexible architectural design, significantly improving its learning performance in complex feature spaces. The TCN-QV time series prediction model is illustrated in Fig 1.

## 3 Methodology

### 3.1 Sliding window block

The traditional full sequence processing method requires loading the entire sequence at once, which is often impractical. This approach demands extremely high computational resources as it necessitates performing calculations on the entire sequence. Consequently, computational complexity increases linearly with the length of the sequence. To address the computational and memory challenges posed by long time series inputs, a sliding window mechanism was implemented for data processing. By defining a fixed-size window, the focus can be placed on the relationships between local time steps while avoiding the high overhead associated with processing the entire sequence at once. This method effectively extracts local features while preserving the flow of global information.

**Fig 2:**
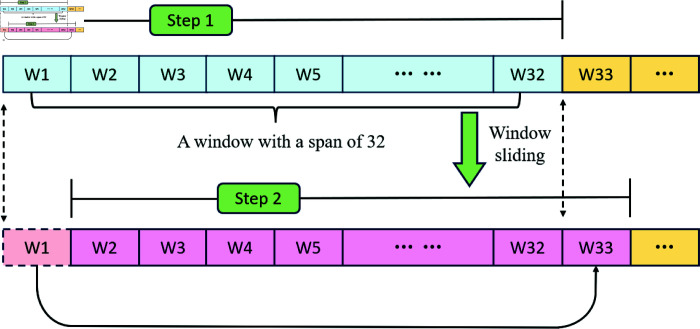
Sliding window diagram.

The initial window drawing concept of the model is illustrated in Fig 2. Through this local sliding window strategy, the model achieves efficient feature extraction at lower time steps, significantly reducing computational complexity while retaining key global contextual information for the time series. This approach enables the model to learn and capture useful patterns in the data more effectively.

### 3.2 Query attention block

This code integrates the QV attention mechanism with the TCN module to achieve efficient feature extraction and modeling. The initial input data is passed through a Dilated Causal Convolution (DC-Conv) layer, which performs dilated causal convolution operations to capture local features within the time series. The output is then projected through a linear layer to generate query (Q) and values (V), which are subsequently processed using a self-attention mechanism. Queries are combined with values through element-wise multiplication to enhance the model’s ability to focus on important features.

To address issues related to gradient vanishing and network degradation, the expressive capability and stability of the model are enhanced through the incorporation of residual connections throughout the entire architecture. This enables effective collaboration between the attention mechanisms and TCN, allowing the model to capture complex patterns and long-range dependencies in the data.

#### 3.2.1 TCN attention.

Compared to traditional RNNs, one-dimensional convolutional networks offer more stable gradient propagation, reducing problems related to gradient vanishing or exploding and providing strong parallel computing capabilities [29]. However, the convolutional layer, specifically Causal Convolution (C-Conv), is still limited by the local receptive field during the convolution process, making it insufficient for modeling longer sequences and recognizing long-term dependencies [30].

To extract more comprehensive temporal information, using a larger receptive field or increasing network depth can improve feature extraction. However, these modifications significantly increase the training time of the network and are often accompanied by overfitting. This requires the exploration of improved model architectures to address these challenges.

**Fig 3:**
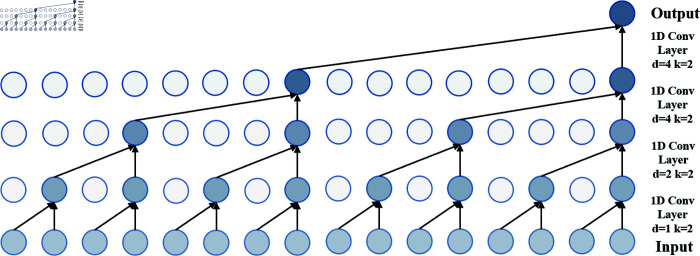
Computational process of DC-Conv.

To alleviate these issues, C-Conv can be effectively improved to DC-Conv, which expands the receptive field by enlarging the convolution kernel. The specific implementation is illustrated in Fig 3. This approach achieves the expansion of the convolution kernel without increasing the network depth, thereby enhancing the model’s ability to process long sequence data more effectively.

The TCN Attention Block presented in this article is primarily designed to process sequential data and capture long-term dependencies in time series. The receptive field is influenced by factors such as dilation rate, kernel size, and network depth. To achieve a more stable extraction effect, both the dilation coefficient is introduced and the network architecture is deepened.

In this module, there are three sub-blocks, each containing convolutional layers, clipping layers, ReLU activation functions, and dropout layers. These are encapsulated into a single TCN block. Subsequently, both the dilation coefficient and causality coefficient are incorporated to expand the convolutional kernel, thereby increasing the model’s receptive field and enhancing its capability to process longer sequences. In this context, *y*[*x*] represents the output of the convolution operation, *w*[*m*] denotes the weight of the convolution kernel, *x* [ *t* − *d* ⋅ *m* ]  is the element of the input sequence, and d is the dilation rate. The calculation equation is presented in Eq (1).

$$\begin{array}{ccc} y[x]=\overset{k-1}{\underset{m=0}{\sum }}w[m]\cdot x[t-d\cdot m] & & \end{array}$$ (1)

At the same time, to avoid gradient vanishing or network degradation, these layers are stacked and connected to residuals, enabling the TCN to capture features at different time scales and fuse them together. The activation function RELU used in the module is shown in Eq (2). The specific calculation equations are presented in Eq (3). Eq (4) summarizes the stacking of convolutional layers and the residual connections in the TCN.

$$\begin{array}{ccc} \text{ReLU}(x)=\max(0,x) & & \end{array}$$ (2)

$$\begin{array}{ccc} f(x)=\text{ReLU}(Weight_{\text{Norm}}(y[x])) & & \end{array}$$ (3)

$$\begin{array}{ccc} {\text{output}}_{\text{TCN}}=\text{TCN}(x)=\text{dropout}(f(x)),\;n\in \{1,2,3\} & & \end{array}$$ (4)

#### 3.2.2 QV(Query Value) attention.

Compared to traditional sequence processing models such as RNNs and LSTMs [31], self-attention mechanisms are more efficient in capturing long-range dependencies within sequences. This is because the self-attention mechanism calculates the correlation between each element in the sequence and assigns a weight to each element, enabling the model to consider all related elements when processing the current element. Additionally, self-attention mechanisms exhibit strong parallelism, significantly enhancing training speed.

However, the self-attention mechanism has some drawbacks. One major issue is that its computational complexity increases linearly with the length of the sequence, which can lead to significant resource consumption for extremely long sequences [32]. Furthermore, self-attention mechanisms may not be as effective as RNNs or LSTMs for processing short sequences due to their lack of sensitivity to temporal order. This means that in certain tasks, self-attention mechanisms may not fully utilize the temporal information present in the sequence.

Although the traditional self-attention mechanism in Transformers effectively captures long-range dependencies through the QKV structure, it may introduce redundancy for long sequences and show lower sensitivity for short sequences. Therefore, this article replaces the traditional self-attention mechanism with an improved QV (Query Value) Attention mechanism.

Compared to traditional self-attention, QV Attention removes the Keys component from the self-attention mechanism, resulting in the loss of the ability to process contextual relationships. However, by reducing the computational processes associated with Queries (Q) and Keys (K) in traditional self-attention mechanisms, the computational and processing efficiency of the model is improved. The QV attention module primarily calculates the self-attention weights within a sequence to capture correlated information. In this module, the model receives the output from the TCN module, and the data processing steps are illustrated in Eqs (5), (6), and (7).


$$\begin{array}{ccc} Z^{\prime }={\text{output}}_{TCN}.\text{permute}(0,2,1) & & \end{array}$$ (5)

$$\begin{array}{ccc} Q=Z^{\prime }W_{Q}+b_{Q} & & \end{array}$$ (6)

$$\begin{array}{ccc} V=Z^{\prime }W_{V}+b_{V} & & \end{array}$$ (7)

Meanwhile, this class incorporates a self-attention mechanism that allows the model to simultaneously focus on information from multiple time steps at different positions. Both the Q and V matrices have dimensions of (seq_length,batch_size,hidden_size). The output calculation equation is shown in Eq (8).


$$\begin{array}{ccc} \text{output}=Q⊙V & & \end{array}$$ (8)

Subsequently, compared to the traditional Transformer structure, this model constructs a residual connection between the input and output. Residual connections enable the direct propagation of gradients back to the original input, thereby avoiding the problems of vanishing or exploding gradients. This characteristic helps achieve fast convergence of deep networks while simplifying the optimization process, as only the gradient in the residual part needs to be considered. Consequently, this enhances gradient propagation, accelerates training, and improves the model’s generalization ability.

To further enrich the expressive power of the model, the output undergoes a nonlinear transformation through two MLPs, and dropout is introduced to prevent overfitting [33]. Ultimately, the model maps the output to the desired final output dimension. Additionally, to enhance the adaptive ability of the model, residual connections are constructed to maintain information flow.

### 3.3 MLP block

In traditional Transformer architectures, self-attention mechanisms are commonly used to process sequential data. Although the self-attention mechanism is excellent at capturing global dependencies, its effectiveness in handling complex nonlinear patterns and feature interactions is limited by linear transformations, which can negatively affect the model’s applicability and performance in diverse tasks. The MLP layer introduced in this study addresses these limitations, enhancing the model’s feature extraction and nonlinear pattern recognition capabilities [34]. This MLP layer consists of two linear transformations and a Gaussian Error Linear Unit (GELU) [35] activation function.

Although traditional ReLU activation functions are favored for their computational simplicity and fast convergence, they possess a significant limitation: negative input values yield zero output and a zero first derivative, which can lead to the phenomenon of "dead neurons." These inactive neurons impede parameter updates during training, diminishing the network’s plasticity and constraining its capacity to learn complex patterns.

To overcome these limitations, the GELU serves as a viable alternative. Built upon the Gaussian error function, GELU permits non-zero gradients for negative input values, facilitating continuous learning and parameter updates within neurons. Consequently, GELU frequently surpasses ReLU in tasks that require fine feature extraction and complex pattern recognition.

Therefore, compared to traditional self-attention mechanisms, the MLP layer in this model can better simulate complex nonlinear relationships by using the GELU activation function. The introduction of the GELU activation function not only improves the model’s expressive power but also enhances its adaptability to complex data distributions. The GELU calculation equation is presented in Eq (9).


$$\begin{array}{ccc} \text{GeLU}(x)=x*P(X\leq x)=x*\Phi (x)=x\int_{-\infty }^{x}\frac{e^{-\frac{{\left(X-\mu \right)}^{2}}{2\sigma^{2}}}}{\sqrt{2\pi }\sigma }dX & & \end{array}$$ (9)

The model maps the input from the hidden layer dimension $d_{\text{model}}$ to the extended dimension $d_{\text{ff}}$, and then maps it back to $d_{\text{model}}$. This design not only enhances the representation ability of the model, enabling it to capture complex nonlinear relationships, but also promotes stronger learning of local and global dependencies within the data through effective feature aggregation.

### 3.4 Long-term prediction framework

Long-term forecasting not only requires models to capture short-term patterns but also to accurately predict future behaviors and trends, which is particularly important in the field of financial analysis. Effective long-term forecasting can provide a foundation for decision-making, reduce risks, and optimize resource allocation.

The classic TCN architecture effectively captures contextual features through the DC-Conv module to achieve long-term prediction. In contrast, RNN models, such as LSTM and GRU, employ gating mechanisms for long-term prediction. Given the differences in network structures, it is crucial to select the most appropriate prediction method for long sequence forecasting and quantify the prediction results.

**Fig 4:**
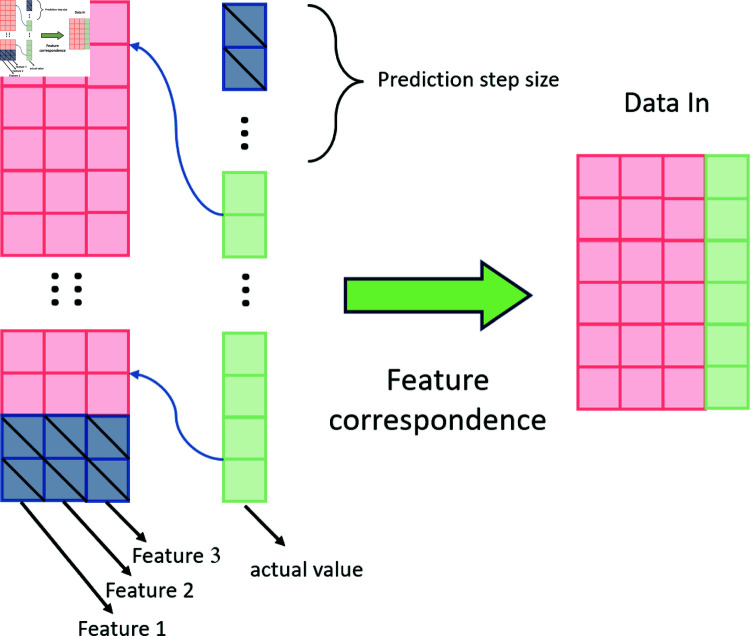
Feature data misalignment processing.

In this article, a long-term prediction method is implemented as shown in Fig 4. To achieve long-term prediction, the features and the true values were misaligned and made to correspond one by one. To ensure accurate extraction of contextual relationships by the model, the TCN model was selected for the initial stage of data processing. However, it has been observed that although TCN demonstrates better overall accuracy in data prediction compared to RNN models, its prediction results exhibit greater volatility, and the model encounters challenges in identifying key nodes of price changes in long-term forecasting. To reduce the volatility of prediction results and enhance the model’s performance, the QV Attention module was introduced.

**Fig 5:**
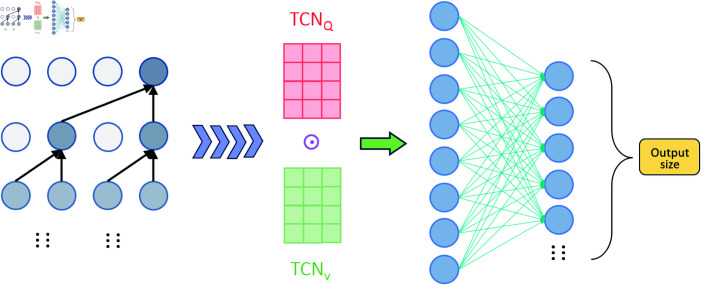
Feature data misalignment processing.

This integration enables the TCN’s long-term prediction method to fully utilize the advantages of QV Attention in long sequence forecasting, as shown in Fig 5. This approach can more accurately predict long sequences, thereby fully exploiting the model’s potential.

## 4 Experimental section

### 4.1 Experimental environment

The server used for this experiment runs on a 64-bit Windows 11 system. It is equipped with a 12th generation Intel Core i7-1260P CPU with a clock speed of 2.10 GHz. The development tool utilized in this experiment is PyCharm 2020.1.3, and the programming language is Python 3.8.10.

### 4.2 Experimental dataset

The dataset chosen for this experiment consists of trading data for Shanghai gold in China, spanning from October 30, 2002, to July 24, 2024. The gold samples in the dataset include purities of 99.99% and 99.95%. Additionally, the experiment involves trading data for 100 grams of gold in Shanghai from December 25, 2005, to July 24, 2006, as well as trading data for Shanghai Gold (T+D) from September 27, 2004, to July 24, 2024. The data was obtained from the CBC Metal Mesh platform website through a third-party data interface.

The daily trading data encompasses various parameters, such as the opening price, highest price, lowest price, and closing price of Shanghai Gold Futures. Notably, the price of gold in Shanghai has risen steadily from approximately 85 yuan to around 550 yuan between 2002 and 2024, showing significant fluctuations during this upward trend, which complicates predictive modeling. Table 1 presents a selection of gold price data in Shanghai. In this study, the selected features are date, opening price, highest price, and lowest price, with the prediction target being the closing price.

In Table 1, the columns are defined as follows:

**Date**: Represents the transaction time.

**Open**: Indicates the daily opening price of the Shanghai Gold industry in China.

**Highest**: Signifies the highest price of Shanghai gold for that day.

**Lowest**: Denotes the lowest price of Shanghai gold for that day.

**Close**: Represents the closing price of Shanghai gold for that day.

**Table 1:** Shanghai gold price data table.

Date	Product	Open	Highest	Lowest	Close
2019/11/01	Gold	343.5	343.5	342.69	342.83
2019/11/04	Gold	343.21	343.21	342.20	342.30
2019/11/05	Gold	341.9	341.9	340.00	340.29
2019/11/06	Gold	336.5	336.5	334.85	335.73
2019/11/07	Gold	337.0	337.5	336.9	337.43
2019/11/08	Gold	343.8	343.8	329.6	331.04

### 4.3 Data preprocessing

This study selects the top 80% of the dataset as the training set and the bottom 20% as the testing set. To prevent the excessive influence of certain feature data on model training, normalization is employed. The training and testing datasets are normalized separately by using the Min-Max scaling method, which scales all data to the range [0,1]. This approach helps balance weight updates and promotes more effective model training by reducing the impact of differences in the order of magnitude between features.The scaling calculation equation is presented in Eq (10).


$$\begin{array}{ccc} x^{\prime }=\frac{x-\min(x)}{\max(x)-\min(x)} & & \end{array}$$ (10)

The dataset can be represented as $S=\{x_{1},x_{2},x_{3},x_{4},\ldots ,x_{n}\}$, where *n* is the number of data entries. *x* is a d-dimensional vector that represents various price data of the Shanghai gold market in China on a given day. By applying a sliding window technique to partition the data into a more detailed structure, the adjusted format can be denoted as follows:$X_{t}=\{x_{t-s},x_{t-s+1},\ldots ,x_{t-1}\}$.Here, $X_{t}$ represents the window centered at the time point *t*, and *s* signifies the size of the time window. Consequently, the constructed window includes the input data from the nearest *s* time steps.

**Fig 6:**
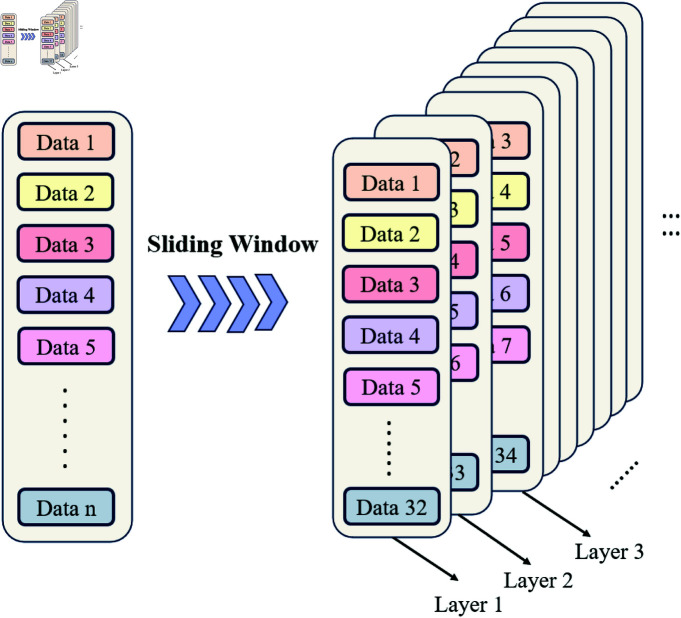
Time sequence construction process.

Thus, the dimensionality of the dataset is  ( *n* , *d* ) . The time series sample set generated is three-dimensional data with dimensions ( *n* , *s* , *d* ) , where *n* denotes the number of time slices, *s* represents the number of time steps in each sample, and *d* corresponds to the number of data items encapsulated in the Shanghai gold pricing data. Assuming that the experimental data length is *n*, Three-dimensional data is created based on a step size of 32 and a dimension *d*. The construction process is illustrated in Fig 6:

The data from rows 1 to 32 constitutes the first layer. The data from rows 2 to 33 forms the second layer, and so on. This leads to a total of n-32 layers. Each data point represents a time step. From the input perspective, it has a dimensionality of *d* = 3, while from the output perspective, *d* = 1. The construction of the three-dimensional data is accomplished through sliding window operations.

To evaluate the performance of various aspects of the model and comprehensively assess its predictive capability, I employ multiple metrics: RMSE (Root Mean Square Error), MAE (Mean Absolute Error), NRMSE (Normalized Root Mean Square Error), MAPE (Mean Absolute Percentage Error), and R2 (Coefficient of Determination). The combination of these metrics enables a multifaceted analysis of the model’s performance, helping to identify potential issues and optimize the model, thereby enhancing its practicality and reliability.

When $y_{i}$ represents the true value, ${ŷ}_{i}$ denotes the predicted value, *n* is the sample size, and *ȳ* is the average of the true values, the calculations for these indicators can be expressed as follows:

RMSE: This metric is computed by calculating the square root of the error between predicted and actual values. It is particularly effective in highlighting the impact of larger errors, especially in highly nonlinear scenarios, thus making it a robust measure of model fit. The indicator is calculated using Eq (11).


$$\begin{array}{ccc} RMSE=\sqrt{\frac{1}{n}\overset{n}{\underset{i=1}{\sum }}{\left(y_{i}-{ŷ}_{i}\right)}^{2}} & & \end{array}$$ (11)

MAE: This metric assesses model performance by determining the average absolute error. It provides a clear understanding of the average deviation between predicted and actual values, thus reflecting the model’s reliability and stability. The indicator is calculated using Eq (12).


$$\begin{array}{ccc} MAE=\frac{1}{n}\overset{n}{\underset{i=1}{\sum }}|y_{i}-{ŷ}_{i}| & & \end{array}$$ (12)

NRMSE: This normalized measure employs RMSE to facilitate comparisons among different datasets.The indicator is calculated using Eq (13).


$$\begin{array}{ccc} NRMSE=\frac{\text{RMSE}}{\max(y)-\min(y)} & & \end{array}$$ (13)

MAPE: This metric evaluates the percentage error of predicted values in relation to actual values. It is suitable for assessing time series data with prolonged variations, effectively demonstrating the performance of relative errors.The indicator is calculated using Eq (14).


$$\begin{array}{ccc} MAPE=\frac{100}{n}\overset{n}{\underset{i=1}{\sum }}\left|\frac{y_{i}-{ŷ}_{i}}{y_{i}}\right| & & \end{array}$$ (14)

$R^{2}$: This value measures the model’s explanatory power with respect to data variability. It has a range from 0 to 1. A value close to 1 indicates a strong explanatory power for the actual data.The indicator is calculated using Eq (15).


$$\begin{array}{ccc} R^{2}=1-\frac{\overset{n}{\underset{i=1}{\sum }}{\left(y_{i}-{ŷ}_{i}\right)}^{2}}{\overset{n}{\underset{i=1}{\sum }}{\left(y_{i}-ȳ\right)}^{2}} & & \end{array}$$ (15)

## 5 Experimental result

### 5.1 Prediction experiment

In this experiment, a horizontal comparison was conducted to validate the advantages of integrating the TCN with the QV Attention model. This study involved evaluating seven prominent time series prediction models: LSTM, GRU, TCN, Transformer, MLP, TCN-LSTM, and Informer [36]. By assessing the performance of these models on the same dataset, we can perform a comprehensive analysis of each model’s predictive capabilities in practical applications, thereby providing valuable insights into their respective strengths and weaknesses.

**Fig 7:**
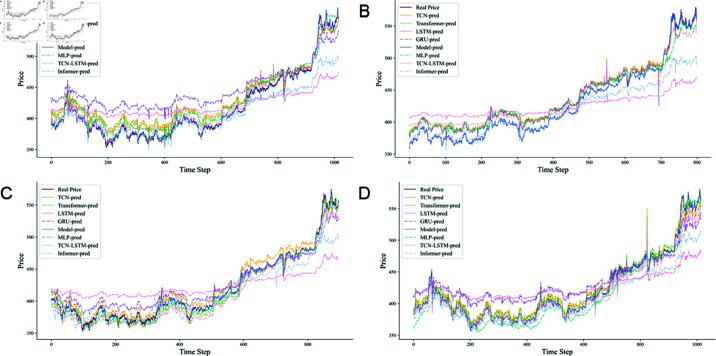
Prediction results of different models in different datasets. (A) Au99.99% prediction result. (B) Au99.95% prediction result. (C) Au100g prediction result. (D) Au(T+D) prediction result.

Four datasets were used for training and evaluation, with each model undergoing meticulous adjustments to optimize performance for specific tasks. Subsequently, the results of these models are compared with the experimental outcomes of the TCN-QV model. This horizontal comparison allows us to identify the advantages of combining the TCN with the QV Attention model, providing a strong empirical foundation for future research. The experimental results for the different datasets in this study are presented in Table 2, while the predicted outcomes are illustrated in Fig 7.

**Table 2:** Experimental results of different models in different datasets.

Dataset	Model	RMSE	MAE	NRMSE	MAPE	R^2^
Au99.99%	LSTM	35.8863	30.0408	0.1609	7.0509	0.5363
	GRU	34.9688	31.9181	0.1568	8.0561	0.5597
	TCN	16.5757	15.3074	0.0743	3.8330	0.9010
	Transformer	13.3704	12.1901	0.0599	3.0491	0.9356
	MLP	13.7417	11.3427	0.0616	2.7765	0.9320
	TCN-LSTM	9.1289	7.3752	0.0409	1.8209	0.9699
	Informer	20.8485	12.5161	0.0935	2.6154	0.8435
	TCN-QV	1.8828	1.5556	0.0084	0.3887	0.9987
Au99.95%	LSTM	34.4471	29.0156	0.1541	6.7664	0.5724
	GRU	23.1681	20.5721	0.1036	5.0673	0.8066
	TCN	8.6572	7.2393	0.0387	1.7543	0.9729
	Transformer	7.0826	5.7303	0.0316	1.3764	0.9819
	MLP	14.1362	11.0006	0.0632	2.6128	0.9275
	TCN-LSTM	8.5836	5.9419	0.0384	1.3052	0.9732
	Informer	20.7045	14.8829	0.0926	3.2272	0.8445
	TCN-QV	0.3094	0.2773	0.0013	0.0640	0.9999
Au100g	LSTM	40.8384	33.9493	0.1846	7.6876	0.4850
	GRU	12.8127	11.6128	0.0579	2.8701	0.9493
	TCN	14.7436	13.5063	0.0666	3.3384	0.9328
	Transformer	12.9643	11.4634	0.0586	2.8563	0.9481
	MLP	15.7926	13.5906	0.0713	3.2934	0.9229
	TCN-LSTM	16.1558	14.0682	0.0730	3.4387	0.9194
	Informer	22.3190	13.5343	0.1008	2.7765	0.8461
	TCN-QV	0.3217	0.2556	0.0014	0.0597	0.9999
Au(T+D)	LSTM	35.1455	30.2365	0.1605	7.1352	0.5318
	GRU	14.7899	12.9250	0.0675	3.2031	0.9170
	TCN	8.9249	7.8694	0.0407	1.8793	0.9698
	Transformer	6.9976	5.9422	0.0319	1.4544	0.9814
	MLP	10.2153	7.1664	0.0466	1.6937	0.9604
	TCN-LSTM	10.6502	9.0360	0.0486	2.1923	0.9570
	Informer	18.8461	13.2170	0.0860	2.9707	0.8653
	TCN-QV	1.1042	0.9543	0.0050	0.2382	0.9995

When comparing the prediction results of different models, the performance of the TCN-QV model is particularly remarkable. The difference between its predicted values and the true values is extremely small—almost negligible—as demonstrated by the high degree of overlap between its predicted curve and the true value curve. This outstanding consistency indicates that the TCN-QV model excels at capturing trends and details within the data, accurately reflecting fluctuations and changes.

In contrast, models such as LSTM and Informer exhibit relatively inferior performance. While they are capable of recognizing the volatility in future data, they struggle to effectively represent the magnitude of this volatility. The TCN-LSTM model, which incorporates TCN to identify temporal correlations in the data, shows significant improvement over the traditional LSTM, although it still experiences some degree of error.

The prediction curves generated by MLP, TCN, GRU, and Transformer show relatively small deviations from the actual values. However, notable discrepancies remain in areas with significant data fluctuations, leading to considerable prediction errors. For instance, while the TCN model’s predictions are closer to the true values, it still experiences significant volatility.

In this experiment, the TCN-QV model not only outperformed other models across various performance indicators but also exhibited greater accuracy and stability in its predictions. This clearly highlights the potential and advantages of the TCN-QV model for time series prediction tasks, providing strong support for future research and applications.

### 5.2 Processing latency experiment

In contrast, different models exhibit varying testing speeds during the iteration process. This experiment compares and tests commonly used models (including TCN, LSTM, GRU, and Transformer) under consistent conditions, ensuring the same test dataset and approximately the same training parameters (with about 84,300 parameters). By statistically analyzing their testing times, we can evaluate the models’ ability to generate outputs after learning from the dataset, which has broader practical implications.

**Fig 8:**
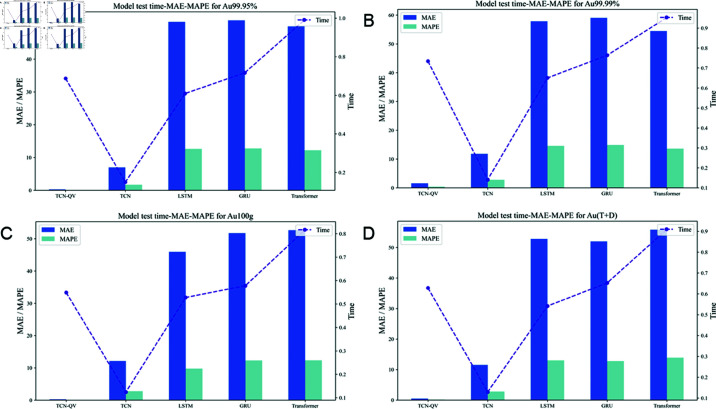
Experimental results of latency in different datasets. (A) Model test time-MEA-MAPE for Au99.95%. (B) Model test time-MEA-MAPE for Au99.99%. (C) Model test time-MEA-MAPE for Au100g. (D) Model test time-MEA-MAPE for Au(T+D).

For the four existing datasets, the testing duration and prediction metrics of each model are presented in Fig 8. In this figure, the line represents the testing duration, while the bar chart shows the metrics used to assess the models’ prediction results.

As is evident from Fig 8, the testing time of the TCN model is relatively short compared to that of the other models, indicating its high efficiency in data processing. Closely following is the TCN-QV model used in this study. It has a testing duration similar to that of LSTM but generally lower than that of the GRU model, demonstrating its outstanding efficiency on these datasets. Conversely, the Transformer model exhibits the longest computation time, likely due to its complex structure and computational requirements.

When evaluating the accuracy of the models, the graph shows that both the TCN-QV and TCN models exhibit relatively low MAE and MAPE values compared to others. Specifically, the TCN-QV model achieves significantly lower values than the TCN model. In contrast, both LSTM and GRU models present high error metrics, particularly in MAE. The Transformer model also shows elevated values, indicating that the predictive capabilities of LSTM, GRU, and Transformer models were not effective in this experiment.

In summary, although the testing time of the TCN-QV model is longer than that of the TCN model, the results indicate that the model’s accuracy has significantly improved compared to TCN. Furthermore, while its testing duration is similar to that of LSTM, its performance is notably superior. The same holds true for the comparison between GRU and Transformer. Therefore, the TCN-QV model presented in this study has substantial practical application value.

### 5.3 Time step experiment

**Fig 9:**
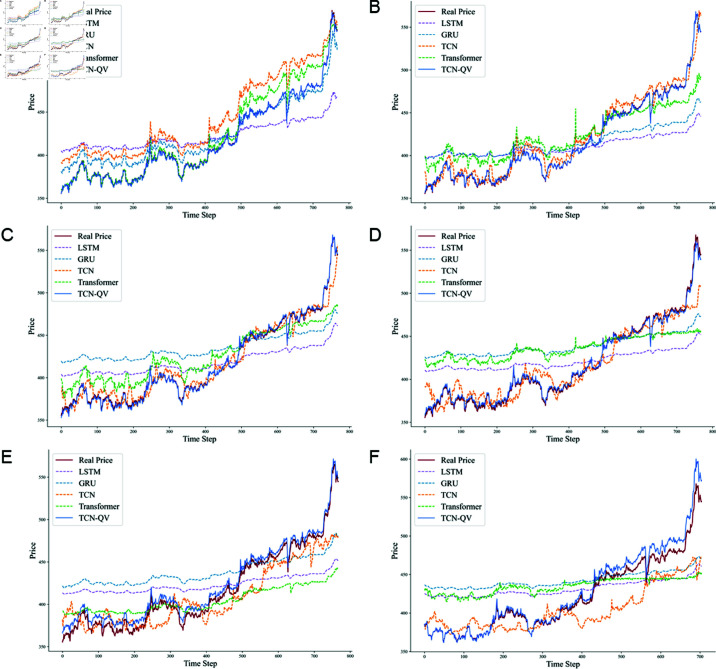
Prediction results of different models in different datasets. (A) Predict 1 future time point. (B) Predict 8 future time point. (C) Predict 16 future time point. (D) Predict 32 future time point. (E) Predict 64 future time point. (F) Predict 128 future time point.

In this experiment, I aim to evaluate and compare the impact of predicting different time series lengths on model performance, including our proposed model and several existing models. The models employed in this study include LSTM, GRU, TCN, and Transformer. These models have demonstrated good performance in processing time series data; however, their efficacy may vary when predicting different lengths of time series. Time series data is selected from the Au (T+D) dataset and segmented based on the predictive time step training data, ensuring that the training data aligns with the predicted data across multiple time steps. Subsequently, this data is divided into subsequences of varying lengths for model training and testing. Specifically, multiple time series lengths are established, and experiments are conducted for each length. Each model is trained on the same training and test sets to ensure fairness in the experiment. The experimental results are presented in Table 3, and the predicted curves are shown in Fig 9.

**Table 3:** Experimental results of different time steps in Au(T+D).

Prediction step	Model	RMSE	MAE	NRMSE	MAPE	R^2^
1	LSTM	32.2433	28.4518	0.1518	6.8324	0.5228
	GRU	14.1026	12.1669	0.0664	3.0552	0.9087
	TCN	28.3761	27.58	0.1336	6.676	0.6304
	Transformer	11.74	8.3543	0.0553	1.8663	0.9367
	TCN-QV	0.3662	0.27	0.0017	0.0636	0.9999
8	LSTM	35.9655	28.9333	0.1694	6.6718	0.4063
	GRU	30.7447	25.7554	0.1448	6.0382	0.5661
	TCN	10.6777	8.4813	0.0503	2.0192	0.9477
	Transformer	22.6993	18.7441	0.1069	4.5382	0.7635
	TCN-QV	0.471	0.4138	0.0022	0.1012	0.9998
16	LSTM	33.0192	28.2534	0.1555	6.6804	0.4996
	GRU	37.3655	32.8811	0.176	8.162	0.3592
	TCN	12.2414	8.7511	0.0576	2.0931	0.9312
	Transformer	22.4864	18.3579	0.1059	4.4522	0.7679
	TCN-QV	1.1312	1.0020	0.0053	0.2465	0.9994
32	LSTM	36.8615	32.1598	0.1736	7.6726	0.3763
	GRU	41.4633	36.3063	0.1925	9.0589	0.2109
	TCN	16.2962	11.3135	0.0767	2.6882	0.8781
	Transformer	39.8636	34.7048	0.1877	8.5745	0.2706
	TCN-QV	1.9487	1.6541	0.0091	0.3997	0.9982
64	LSTM	38.0814	33.5021	0.1793	8.0299	0.3344
	GRU	33.492	33.5902	0.1813	8.3950	0.3199
	TCN	21.7834	17.0363	0.1026	3.9726	0.7822
	Transformer	34.9097	25.5725	0.1644	5.7337	0.4406
	TCN-QV	8.894	6.6873	0.0324	1.6618	0.9781
128	LSTM	38.0892	33.6572	0.1854	8.1220	0.3317
	GRU	42.9069	34.7662	0.2074	9.1641	0.1451
	TCN	21.7834	17.036	0.1036	4.2106	0.8833
	Transformer	40.6482	34.5109	0.1973	8.9323	0.2962
	TCN-QV	9.0081	6.0436	0.0438	1.2932	0.9626

When predicting shorter time steps, the TCN-QV model shows significantly better performance than the other models. Its RMSE and MAE values are notably lower than those of the LSTM and GRU models. The figure illustrates that the predicted curve (dark blue) of TCN-QV almost completely overlaps with the actual price curve, highlighting its advantage in short-term forecasting. As the predictive time step increases, the performance of the TCN and Transformer models gradually improves. Particularly when the time step is 16, TCN’s RMSE and MAE show strong performance, demonstrating its potential for mid-term prediction. Although the predictive performance of the TCN-QV model declines somewhat, it still maintains extremely high accuracy compared to the other models, retaining its status as the overall top performer.

As the predicted time steps extend to longer durations, the prediction accuracy of all models generally decreases. Notably, the RMSE and MAE metrics for LSTM and GRU models significantly increase. The TCN model shows errors in predicting the overall trend, highlighting its limitations in long-term forecasting. Although TCN-QV experiences a slight downward trend, its prediction results remain more accurate than those of the other models.

From the provided prediction in Fig 9, it is evident that the disparity between the actual price curve and the predicted curves of each model varies at different time steps. The TCN-QV model demonstrates the highest degree of overlap between its predicted curve and the actual price curve, underscoring its superior predictive ability. In contrast, the prediction curves for LSTM and GRU experience significant fluctuations over long time steps, showing considerable deviations from the true prices and indicating their instability in long-term forecasting.

In summary, the TCN-QV model performs exceptionally well across all time steps, particularly in short-term forecasting. LSTM and GRU offer average performance in short-term scenarios but experience significant declines in long-term forecasting. Consequently, it is apparent that the TCN-QV model demonstrates robust predictive capabilities in price forecasting, especially in scenarios that demand high-precision short-term predictions.

### 5.4 Model ablation experiment

The contextual correlation mechanism of this experimental model is constructed by combining DC-Conv and QV Attention. Therefore, it is essential to investigate the contributions of the attention mechanisms from both components to the model’s performance. Conducting ablation experiments is crucial for evaluating the contributions of the QV Attention and TCN components. By systematically removing or replacing specific components while keeping relevant parameters constant, we can observe changes in model performance and clearly identify the impact of each component on the final results. The results of the ablation experiment are presented in Table 4.

**Table 4:** Model performance metrics.

Dataset	Model	RMSE	MAE	NRMSE	MAPE	R^2^
Au99.99%	TCN	24.965	22.6139	0.1139	5.6685	0.7374
	QV	15.7394	14.4326	0.0718	3.6395	0.8956
	TCN-QV	1.8815	1.5473	0.0086	0.3899	0.9985
Au99.95%	TCN	10.9299	8.3998	0.0499	2.0308	0.9494
	QV	5.8056	4.0636	0.0265	0.9905	0.9857
	TCN-QV	0.3049	0.2731	0.0014	0.0636	0.9999
Au100g	TCN	23.9588	21.6636	0.1083	5.2763	0.8227
	QV	13.2854	11.8225	0.0601	2.929	0.9455
	TCN-QV	0.3218	0.2557	0.0015	0.0598	0.9999
Au(T+D)	TCN	10.4792	7.8697	0.0479	1.8997	0.9584
	QV	4.7771	3.4019	0.0218	0.8072	0.9914
	TCN-QV	0.6512	0.495	0.003	0.126	0.9998

The TCN-QV Attention model significantly outperforms traditional time series prediction models across four datasets. In the Au99.99% dataset, it achieves exceptionally high accuracy, with RMSE and MAE metrics approaching zero and R^2^ values reaching 0.9985, indicating an almost perfect fit.

**Fig 10:**
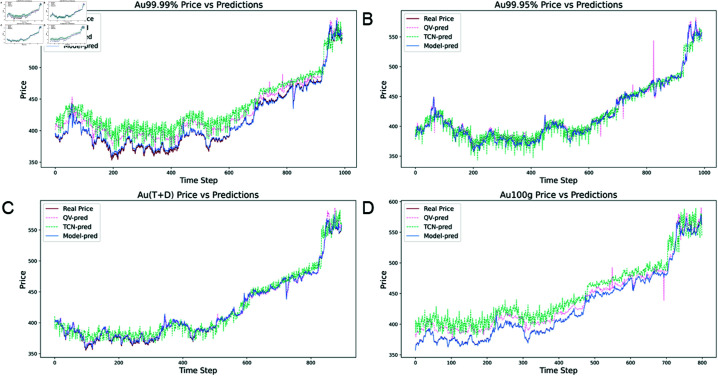
Model prediction results of different datasets in ablation experiments. (A) Au99.99% model prediction results. (B) Au99.95% model prediction results. (C) Au(T+D) model prediction results. (D) Au100g model prediction results.

Conversely, while the TCN model demonstrates better performance than traditional RNN or CNN models, its results remain insufficient, with relatively high RMSE and MAE values indicating notable decreases in accuracy. The QV Attention model also lags behind the TCN-QV Attention model in accuracy, despite some improvements. As shown in Fig 10, the predicted curve of the TCN-QV Attention model nearly overlaps with the actual gold price trend, especially during sharp fluctuations. It excels at capturing these dynamic changes, maintaining high accuracy in both stable and volatile conditions.

The ablation experiment further confirms the TCN-QV Attention model’s advantages in time series prediction tasks, effectively combining the long-term dependency capabilities of time convolutional networks with the QV Attention mechanism. This approach enhances the model’s focus on critical time points, resulting in significant improvements in accuracy and stability.

## 6 Discussion

The experimental results indicate that the TCN-QV model exhibits strong performance in predicting gold prices. For small samples of metal price time series, the optimal TCN-QV model achieved a minimum improvement of approximately 5.47% and a maximum improvement of about 33.69% in MAE compared to the benchmark models across four experimental datasets. Notably, even with an increase in prediction step size, the model continues to maintain good performance. Furthermore, while controlling for the number of training parameters, the TCN-QV model demonstrates relatively high efficiency compared to the recurrent neural network benchmark models, despite its testing time being slower than that of TCN. However, the experiments presented in this paper have certain limitations. Due to the small sample size of the selected gold price dataset, recurrent neural networks like LSTM and GRU exhibit poor predictive performance, while convolutional networks, particularly TCN, perform effectively. Consequently, this study did not explore the predictive performance of TCN-QV on larger datasets, which may impact the model’s generalization ability. Additionally, the training dataset is based on weekday gold price data, which is limited in size, potentially affecting the model’s applicability to larger datasets. The experiments in this study were conducted using CPU computation, which may introduce some errors in time measurement. In the experiments focused on processing latency, we adjusted the training parameters to be similar across models, but we did not ensure complete uniformity. Consequently, the experimental results exhibit certain limitations. Overall, the TCN-QV model shows promising results in gold price prediction and could offer valuable insights for investment decisions and national risk management.

## 7 Conclusion

In this article, the TCN-QV Attention model is proposed, which integrates traditional convolutional networks with variants of self-attention mechanisms to efficiently extract temporal features. By stacking DC-Conv layers through residual connections for context time feature extraction, computational speed is improved without sacrificing accuracy. Additionally, a novel QV Attention mechanism is utilized to enhance the extraction of important information. Furthermore, the impact of QV Attention and the number of residual blocks on overall accuracy is discussed, aiming to achieve optimal model performance. Tests conducted across different prediction time steps show that as the time step increases, the performance of other models significantly deteriorates, while the TCN-QV Attention model maintains strong performance in long-term predictions. Thus, the TCN-QV Attention model can act as a versatile solution for various temporal prediction scenarios. In future research, I will focus on optimizing model complexity and testing duration.

## References

[1] Mantegna RN. Hierarchical structure in financial markets. Eur Phys J B. 1999;11(1): 193–7.

[2] Li Y, Du Q. Oil price volatility and gold prices volatility asymmetric links with natural resources via financial market fluctuations: implications for green recovery. Resources Policy. 2024; 88104279. doi: 10.1016/j.resourpol.2023.104279

[3] Esangbedo MO, Taiwo BO, Abbas HH, Hosseini S, Sazid M, Fissha Y. Enhancing the exploitation of natural resources for green energy: an application of LSTM-based meta-model for aluminum prices forecasting. Resources Policy. 2024; 92105014. doi: 10.1016/j.resourpol.2024.105014

[4] Huang Y, Bai Y, Ding L, Zhu Y-J, Ma Y-J. Application of a hybrid model based on ICEEMDAN, Bayesian hyperparameter optimization GRU and the ARIMA in nonferrous metal price prediction. Cybern Syst. 2022; 54(1):27–59. doi: 10.1080/01969722.2022.2055383

[5] Bai S, Kolter JZ, Koltun V. An empirical evaluation of generic convolutional and recurrent networks for sequence modeling. arXiv preprint. 2018. doi: arXiv:1803.01271

[6] Vaswani A. Attention is all you need. Advances in Neural Information Processing Systems: 2017.

[7] Tong J, Zhang Y. A real-time label-free self-supervised deep learning intrusion detection for handling new type and few-shot attacks in iot networks. IEEE Internet Things J. 2024.

[8] Zhang Y, Liang M, Ou H. Prediction of precious metal index based on ensemble learning and shap interpretable method. Comput Econ. 2024; 1–36.

[9] Gao J, Cao Q, Chen Y. Auto-regressive moving diffusion models for time series forecasting. arXiv preprint. 2024.

[10] Luo Z, Guo W, Liu Q, Tse Y. A hybrid prediction model with time?varying gain tracking differentiator in Taylor expansion: evidence from precious metals. J Forecasting. 2022; 42(5):1138–49. doi: 10.1002/for.2935

[11] YAO J. Study on stock index prediction based on arima and information granular svr combination. Oper Res Manag Sci. 2022; 31(5):214.

[12] He Z, Huang J. A novel non-ferrous metal price hybrid forecasting model based on data preprocessing and error correction. Resources Policy. 2023; 86104189. doi: 10.1016/j.resourpol.2023.104189

[13] Barzegar R, Aalami MT, Adamowski J. Coupling a hybrid CNN-LSTM deep learning model with a Boundary Corrected Maximal Overlap Discrete Wavelet Transform for multiscale Lake water level forecasting. J Hydrol. 2021; 598126196. doi: 10.1016/j.jhydrol.2021.126196

[14] Bhoj N, Singh Bhadoria R. Time-series based prediction for energy consumption of smart home data using hybrid convolution-recurrent neural network. Telematics Inf. 2022; 75101907. doi: 10.1016/j.tele.2022.101907

[15] Chen X, Cao L, Cao Z, Zhang H. A multi-feature stock price prediction model based on multi-feature calculation, LASSO feature selection, and Ca-LSTM network. Connect Sci. 2024; 36(1). doi: 10.1080/09540091.2023.2286188

[16] Brauwers G, Frasincar F. A general survey on attention mechanisms in deep learning. IEEE Trans Knowl Data Eng. 2023; 35(4):3279–98. doi: 10.1109/tkde.2021.3126456

[17] Wang C, Chen Y, Zhang S, Zhang Q. Stock market index prediction using deep Transformer model. Expert Syst Appl. 2022; 208118128. doi: 10.1016/j.eswa.2022.118128

[18] Burukanli M, Yumuşak N. TfrAdmCov: a robust transformer encoder based model with Adam optimizer algorithm for COVID-19 mutation prediction. Connect Sci. 2024; 36(1). doi: 10.1080/09540091.2024.2365334

[19] Li C, Qian G. Stock price prediction using a frequency decomposition based GRU transformer neural network. Appl Sci. 2022; 13(1):222. doi: 10.3390/app13010222

[20] Lu W, Li J, Wang J, Wu S. A novel model for stock closing price prediction using cnn-attention-gru-attention. Econ Comput Econ Cybern Stud Res. 2022; 56(3).

[21] Liu J, Zhang B, Zhang T, Wang J. Soybean futures price prediction model based on eemd-nagu. IEEE Access. 2023.

[22] Xu H, Chai L, Luo Z, Li S. Stock movement prediction via gated recurrent unit network based on reinforcement learning with incorporated attention mechanisms. Neurocomputing. 2022; 467214–28. doi: 10.1016/j.neucom.2021.09.072

[23] Guo X, Hua D, Bao P, Li T, Yao N, Cao Y, et al. A short-term electricity price forecasting method based on improved vmd-pso-cnn-lstm. J Electric Power Sci Technol. 2024; 39(2):35–43.

[24] Wang N, Zhao X. Time series forecasting based on convolution transformer. IEICE Trans Inf Syst. 2023; E106.D(5):976–85. doi: 10.1587/transinf.2022edp7136

[25] Zhao Y, Chen J, Shimada H, Sasaoka T. Non-ferrous metal price point and interval prediction based on variational mode decomposition and optimized LSTM network. Mathematics. 2023; 11(12):2738. doi: 10.3390/math11122738

[26] Tri D, Gu A. Transformers are ssms: generalized models and efficient algorithms through structured state space duality. arXiv preprint 2405.21060. 2024.

[27] Li F, Zhou H, Liu M, Ding L. A medium to long-term multi-influencing factor copper price prediction method based on CNN-LSTM. IEEE Access. 2023; 1169458–73. doi: 10.1109/access.2023.3288486

[28] Luo D, Wang X. Moderntcn: a modern pure convolution structure for general time series analysis. The Twelfth International Conference on Learning Representations. 2024.

[29] Mohsin M, Jamaani F. A novel deep-learning technique for forecasting oil price volatility using historical prices of five precious metals in context of green financing – A comparison of deep learning, machine learning, and statistical models. Resources Policy. 2023; 86104216. doi: 10.1016/j.resourpol.2023.104216

[30] Tian J, Shen C, Wang B, Xia X, Zhang M, Lin C. Lesson: multi-label adversarial false data injection attack for deep learning locational detection. IEEE Trans Dependable Secure Comput. 2024.

[31] Jia Y, Lin Y, Yu J, Wang S, Liu T, Wan H. Pgn: the rnn’s new successor is effective for long-range time series forecasting. arXiv preprint. 2024.

[32] Zhang Z, Han Y, Ma B, Liu M, Geng Z. Temporal chain network with intuitive attention mechanism for long-term series forecasting. IEEE Trans Instrum Meas. 2023.

[33] Tian J, Shen C, Wang B, Ren C, Xia X, Dong R, Cheng T. Evade: targeted adversarial false data injection attacks for state estimation in smart grid. IEEE Trans Sustainable Comput. 2024.

[34] Abu-Doush I, Ahmed B, Awadallah MA, Al-Betar MA, Rababaah AR. Enhancing multilayer perceptron neural network using archive-based harris hawks optimizer to predict gold prices. J. King Saud Univ Comput Inf Sci. 2023; 35(5):101557. doi: 10.1016/j.jksuci.2023.101557

[35] Hendrycks D, Gimpel K. Gaussian error linear units (gelus). arXiv preprint; arXiv:1606.08415. 2016. doi: 10.48550/arXiv.1606.08415

[36] Zhou H, Zhang S, Peng J, Zhang S, Li J, Xiong H, Zhang W. Informer: beyond efficient transformer for long sequence time-series forecasting, 2021.

